# Parvovirus B19 infection in children: a comprehensive review of clinical manifestations and management

**DOI:** 10.1186/s13052-024-01831-6

**Published:** 2024-12-18

**Authors:** Silvia Bloise, Enrico Cocchi, Lorenzo Mambelli, Caterina Radice, Federico Marchetti

**Affiliations:** 1 Department of Pediatrics, Santa Maria delle Croci Hospital, AUSL della Romagna, Viale Vincenzo Randi, 5, Ravenna, Ravenna, 48121 RA Italy; 2 Neonatal Intensive Care Unit, AUSL Romagna, Ravenna, Italy; 3 Department of Medical and Surgical Sciences, Alma Mater Studiorum, University of Bologna, Bologna, Italy

**Keywords:** Parvovirus B19, children, Fifth disease, clinical manifestation

## Abstract

Parvovirus B19 (B19V) is a significant pathogen responsible for a wide range of clinical manifestations, particularly in children and pregnant women. While B19V is most commonly recognized as the cause of Fifth disease, a mild erythematous illness in children, its clinical impact extends far beyond this condition. B19V can lead to severe complications, including transient aplastic crisis in individuals with chronic hemolytic anemias, arthralgia, and more severe joint diseases. During pregnancy, B19V infection poses serious risks, such as spontaneous abortion, non-immune hydrops fetalis, and fetal anemia, particularly when infection occurs between 9 and 20 weeks of gestation. Moreover, B19V is associated with a variety of organ system involvements, including cardiac, neurological, hepatic, and renal complications. These manifestations can range from mild to life-threatening, necessitating a broad spectrum of therapeutic approaches, including symptomatic care, immunoglobulins, corticosteroids, and supportive therapies. Despite the significant clinical burden posed by B19V, no specific antiviral treatment or vaccine is currently available, making early recognition and prompt management crucial for improving patient outcomes. This review provides a comprehensive overview of the diverse clinical presentations of B19V infection, with a focus on pediatric and pregnancy-related complications. It underscores the need for ongoing research into targeted therapies and highlights the importance of vigilant clinical management to mitigate the severe consequences of this pervasive virus.

## Background

Parvovirus B19 (B19V) derives its name from its small size and the code assigned to the serum sample from which it was first isolated. It is a DNA virus, primarily transmitted through respiratory secretions, saliva or infected serum [1, 2]. B19V is best known as the cause of Fifth disease, a self-limiting febrile erythematous illness in children [3]; however, the virus exhibits a wide clinical spectrum, affecting various organs and systems, with manifestations ranging from asymptomatic cases to life-threatening infections [4].

The most affected age group comprises school-aged children (4–11 years), with outbreaks predominantly occurring in winter or spring [5]. In recent years, a significant increase in B19V infections has been observed, with studies from Israel [6] and France [7] reporting a fourfold rise in confirmed cases compared to previous epidemics. Given the increased incidence and clinical diversity of B19V, this paper aims to review the most recent evidence regarding the clinical manifestations and potential complications of parvovirus B19 infection in children and pregnancy.

## Natural history of infection, pathogenesis and immune response

B19V displays a strong tropism for human erythroid progenitor cells. It is directly cytotoxic to colony-forming units (CFU-E) and burst-forming units (BFU-E) within the erythroid lineage, mediated through the erythrocyte P antigen, the cellular receptor for B19V. Individuals lacking the P antigen are naturally resistant to infection, as lower levels of this antigen are expressed in a limited number of other non-erythroid cell types [8].

The incubation period is usually 4 to 14 days; approximately four to six days after infection, viremia begins and lasts for about one week. This phase coincides with a temporary arrest of hematopoiesis, affecting lymphocytes, reticulocytes, neutrophils, and platelets sequentially. Recovery is associated with the production of virus-specific IgM antibodies, typically occurring 10 to 12 days post-infection. The IgG response starts after two weeks and is crucial for the clearance of the virus and long-term protection [9]. While the humoral immune response is considered the most important for viral clearance, some studies suggest that the cellular immune response also plays a role [10]. 

The clinical evolution of B19V infection varies widely, ranging from asymptomatic cases to mild symptoms or more severe clinical presentations. The severity of the disease is influenced by the age, hematologic status, and immunologic status of the host. In most cases, symptoms such as fever, headache, chills, myalgia, and itching may appear around eight days after infection. During the IgG response phase, and up to the fourth week after infection, a rash (more common in children) and/or arthralgia (more common in adults) may develop [11]. These manifestations are secondary to immune complex formation. At this stage, patients are no longer contagious, as viremia has cleared. In contrast, immunocompromised individuals, who may not mount a detectable immune response, can remain infectious for a longer period, with measurable levels of the virus persisting in their system [12].

**Fig. 1:**
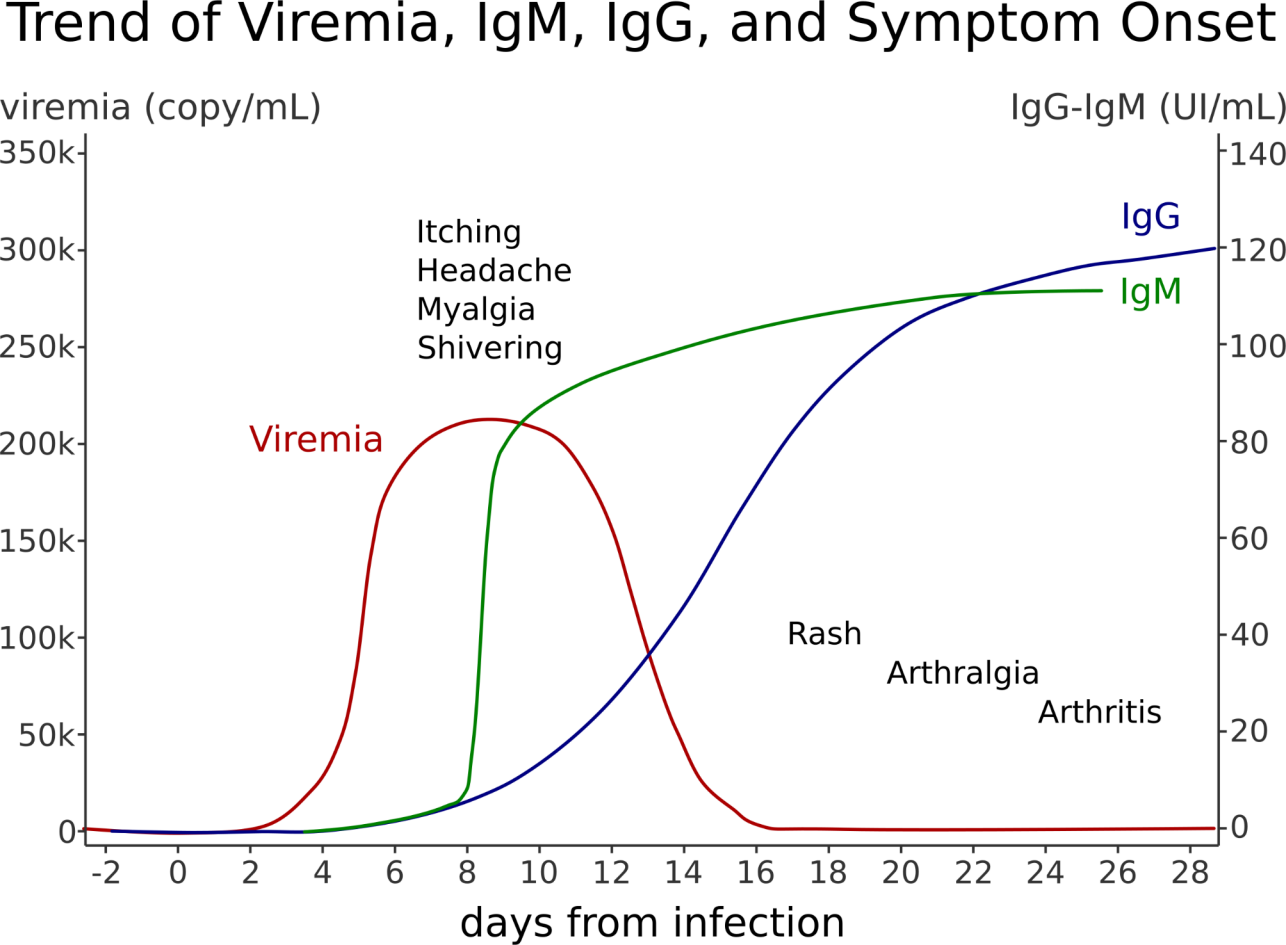
Antibody response to B19V and the related stages of the disease: the first febrile phase, with headache, chill, myalgia, itching; the second phase characterized by rash (more common in children) and/or arthralgia (more common in adults) concomitant with IgG response and contagiousness is exhausted

The Fig. 1 shows the antibody response to B19V and the related stages of the disease.

## Spectrum of clinical manifestations and management of parvovirus B19 infection

**Fig. 2:**
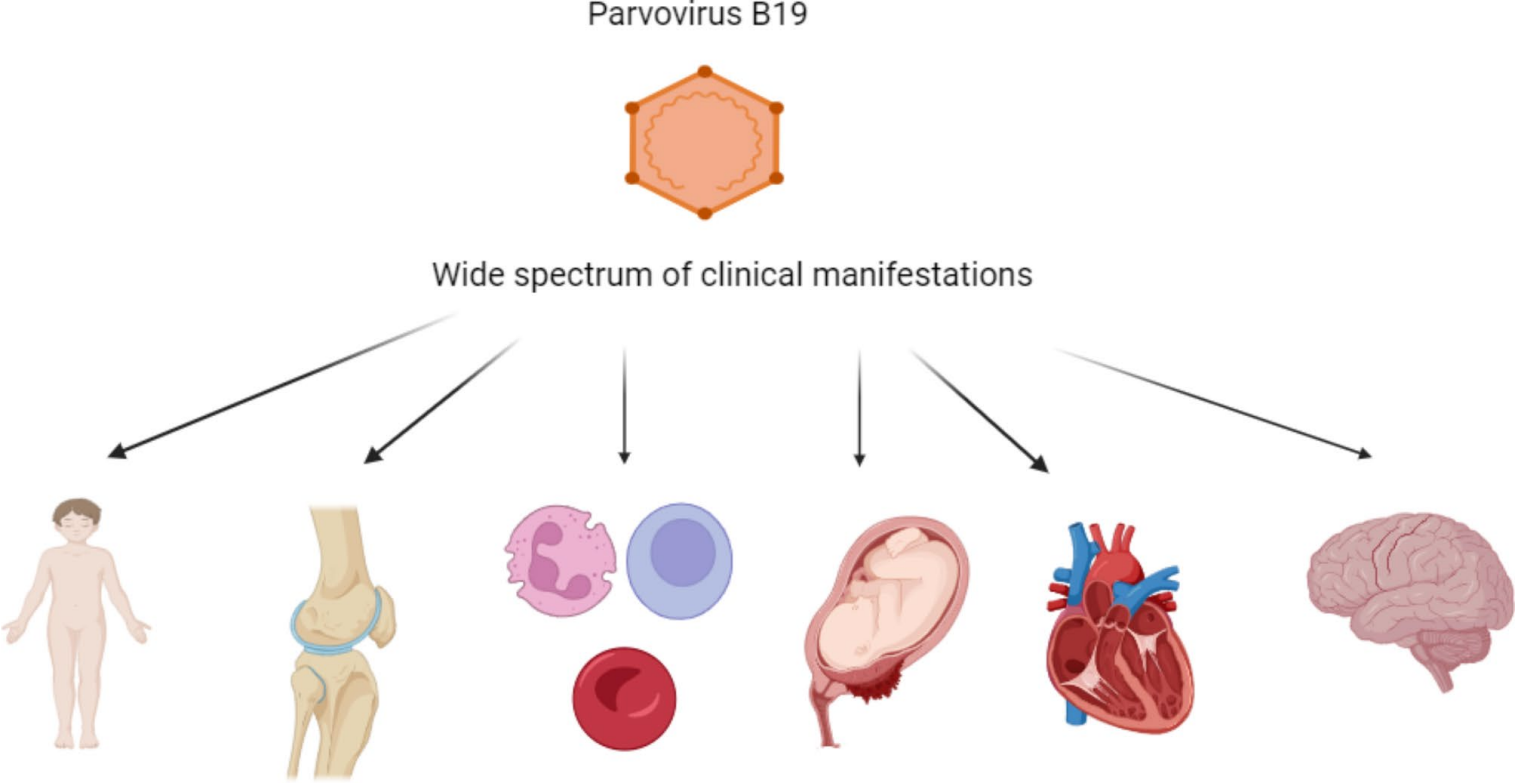
Spectrum of possible clinical features of parvovirus B19 infection. The figure was created by biorender.com

Parvovirus B19 can be responsible for various clinical syndromes (Fig. 2). The possible manifestations of B19V are different and include:

1. **Fifth Disease or Erythema Infectiosum**: This is the most common presentation of B19V infection in children, characterized by a distinctive “slapped cheek” rash, followed by a reticulated or lacy rash on the trunk and limbs.
2. **Joint Manifestations**: B19V can cause arthralgia and acute arthritis, particularly in older children and adults. These manifestations are often self-limiting but can occasionally persist.
3. **Hematologic Manifestations**: B19V is associated with transient aplastic crisis (TAC) in individuals with underlying hemolytic disorders, such as sickle cell disease and hereditary spherocytosis, due to its cytotoxic effect on erythroid progenitor cells.
4. **Cardiac Manifestations**: B19V has been implicated in myocarditis and other cardiac conditions, which can range from mild to severe, potentially leading to chronic cardiac issues such as dilated cardiomyopathy.
5. **Neurological Manifestations**: B19V can affect both the central and peripheral nervous systems, leading to conditions such as encephalitis, meningitis, and Guillain-Barré syndrome.
6. **Manifestations in transplant recipients**: In these patient clinical manifestations of B19V may be atypical and the infection can have severe course.
7. **Other Manifestations**: In addition to the above, B19V can cause other systemic manifestations, including liver and kidney involvement, which are less common but can be severe, particularly in immuno-compromised individuals.

### Fifth disease or erythema infectiosum

“Fifth disease,” also known as “Erythema infectiosum” or “Slapped cheek disease,” is usually a mild, self-limiting illness in children. It is characterized by mild prodromal symptoms, including headache, malaise, and myalgia. The rash typically evolves in three phases. The initial phase is marked by an erythematous rash localized on the cheeks, giving a characteristic slapped appearance, hence the common name “Slapped cheek disease.” In the second phase, the rash becomes macular and spreads to the trunk, extremities, and buttocks as diffuse macular erythema [3]. The third phase is characterized by the fading of the rash, which may leave behind a reticulated or lacy pattern as it clears.

The rash associated with B19V is pruritic in about 50% of cases and is more intense on extensor surfaces, typically sparing the palms and soles. In 80% of cases, the rash assumes a reticulated appearance due to central clearing, a feature that helps distinguish Fifth disease from other exanthematous illnesses. A typical characteristic of the B19V rash is its tendency to recur after various stimuli, such as changes in temperature, exposure to sunlight, exercise, or emotional stress.

In adults, the rash is less characteristic than in children (involving the legs, the trunk, and arms; only the 20% of affected adults have an erythematous rash on the face) and may be mistaken for rubella. Additionally, B19V rash can present in other forms, including morbilliform, confluent, and vesicular patterns. Furthermore, B19V has been observed in association with Gianotti-Crosti syndrome, which features very itchy papulovesicular acrodermatitis [13], and in patients with papular-purpuric “gloves and socks” syndrome, an illness characterized by pruritic, painful acral erythema, fever, and mucosal lesions [14].

There is no specific therapy for the fifth disease. Treatment is symptomatic, with antipyretics for fever and possibly antihistamines if the rash is associated with intense itching.

### Joint manifestations

Parvovirus B19 can cause arthralgias, acute arthritis, and occasionally chronic arthropathy in both children and adults, with joint involvement being more common in adult women [15]. The arthropathy associated with B19V often resembles rheumatoid arthritis, presenting as an acute, symmetric condition that most frequently affects the small joints of the hands, wrists, knees, and feet [16]. Joint stiffness is a common symptom, but unlike rheumatoid arthritis, joint erosion is not observed. Typically, symptoms resolve within a few weeks; however, a minority of patients may develop persistent or recurring arthropathy [17].

Some studies suggest that 20–35% of children with juvenile idiopathic arthritis may show serological evidence of recent B19V infection [18]. However, the association between B19V infection and other childhood rheumatic syndromes remains controversial. Some authors have reported the presence of laboratory markers of inflammation, antibodies against the viral NS1-protein, and B19V genomes in patients with rheumatic diseases, such as juvenile systemic sclerosis and juvenile dermatomyositis, compared to healthy controls [19]. Conversely, other studies do not support a significant relationship between B19V and these conditions. For example, Weissbach et al. [20]. found no difference in the prevalence of B19V-specific IgG antibodies between patients with juvenile idiopathic arthritis and healthy controls.

Currently, there are no clear guidelines for the treatment of chronic parvovirus arthropathy. Nonsteroidal anti-inflammatory drugs (NSAIDs) or short courses of corticosteroids may be effective in managing symptoms. However, given the uncertain significance of detecting viral DNA in joint fluid or tissue, immunoglobulin therapy is not recommended at this time [21].

### Hematologic manifestations

The primary hematologic manifestation of B19V infection is transient aplastic crisis (TAC). Through the blood group P antigen receptor, B19V infects and destroys erythroid progenitor cells, inhibiting erythropoiesis and leading to acute erythroblastopenia and reticulocytopenia. TAC primarily occurs in patients with chronic hemolytic anemias, such as sickle cell disease, hereditary spherocytosis, and thalassemia [22], but it can also occur in patients with decreased blood cell production, such as those with iron deficiency anemia or immunodeficiency [23].

B19V-associated TAC has been well characterized in numerous large case series [24, 25]. In patients with sickle cell disease, aplastic crisis is a serious and life-threatening condition. Parvovirus infection should be suspected in all cases where there is a rapid worsening of anemia accompanied by reticulopenia. Patients with TAC typically present with pallor, weakness, and lethargy due to severe anemia. Potential complications include congestive heart failure, cerebrovascular accidents, and acute splenic sequestration [26]. Laboratory findings typically reveal reticulopenia, a decrease in hemoglobin concentration of more than 30%, and, in some cases, a reduction in white cell and platelet counts [27]. However, it is important to note that B19V infection can be subclinical even in patients with sickle cell disease.

Treatment of TAC is primarily supportive, with blood transfusions indicated for severe anemia until the patient’s immune response can eliminate the infection and red cell production resumes, usually within one to two weeks. Notably, TAC generally occurs only once in immunocompetent patients due to the development of protective immunity [28]. In contrast, in patients with immunodeficiency - whether congenital, secondary to chemotherapy, or related to immunosuppression, especially post-transplantation, or in those with HIV - the virus may continue to replicate, maintaining the aplastic state. This condition is more common in children due to a lack of prior immunity to B19V. In such cases, treatment with immunoglobulin administration should be considered, and repeated courses may be necessary [29, 30]. Common therapeutic regimens include an initial dose of 1–2 g/kg, administered either as a single dose or divided over 2–5 days, followed by a maintenance dose of 0.4 g/kg every 3–4 weeks. The duration of therapy depends on the patient’s clinical response and the recovery of red blood cell production. Another treatment strategy in refractory patients could be the use of the interferon alfa-2a, a therapeutic option tested on adult patients [12].

### Cardiac manifestations

Cardiac manifestations associated with B19V include myocarditis, dilated cardiomyopathy, and isolated left ventricular diastolic dysfunction [31]. B19V is considered one of the most common causes of viral myocarditis. While the exact pathogenetic mechanisms remain unclear, myocarditis could be related to the primary infection via P receptor-mediated virus endocytosis or through autoimmune-mediated inflammation [32].

The most common presenting symptoms of B19V-related myocarditis are tachycardia, tachypnea, fever, and rash. In some cases, myocarditis may present with more severe symptoms such as cardiac arrest, loss of consciousness, and systemic infection, which are associated with a worse prognosis. Abnormal laboratory findings often include elevated levels of troponin, C-reactive protein, B-type natriuretic peptide, white blood cells, and creatinine kinase. However, the absence of these altered laboratory results does not rule out the diagnosis.

Electrocardiogram (ECG) findings frequently show sinus tachycardia, ST-segment changes, prolonged QT interval, T wave inversions, or arrhythmias. Echocardiography is typically the first-line imaging method for suspected myocarditis, though its accuracy is limited due to the lack of specific findings. Common echocardiographic findings include regional wall motion abnormalities (most commonly involving the inferior or inferolateral walls), diastolic dysfunction with preserved left ventricular ejection fraction, and global left ventricular systolic dysfunction.

The gold standard for diagnosing myocarditis is an endomyocardial biopsy, but in clinical practice, cardiac magnetic resonance imaging (MRI) is often used as an alternative. Typical MRI findings include myocardial edema, detected as increased signal intensity on T2-weighted images; hyperemia, corresponding to intense signal in early gadolinium enhancement images; and necrosis or fibrosis, detected on late gadolinium enhancement images. The presence of two out of three criteria supports the diagnosis [33].

There is a strong correlation between myocardial and blood PCR results, with high viral loads being associated with cardiac inflammation [31]. The prognosis for B19V-related myocarditis is variable; some patients recover fully, while others may experience a reduction in left ventricular function and develop dilated cardiomyopathy, potentially requiring surgical intervention [34].

The primary goal of therapy for patients with myocarditis is to manage oxygenation and fluid status. Hemodynamically stable, asymptomatic, or mildly symptomatic patients should be admitted to the hospital for clinical monitoring due to the risk of arrhythmias and heart failure. For patients with heart failure symptoms, the therapeutic focus is on preserving ventricular function and ejection fraction. Treatment typically includes beta-blockers, diuretics, ACE inhibitors, and/or angiotensin-II receptor blockers. In cases of severe and acute decompensating illness, inotropic support and/or mechanical circulatory support may be necessary [35].

Regarding immunotherapy, current recommendations suggest the use of high-dose intravenous immunoglobulins (IVIG) for patients with B19V-related myocarditis (33, 36–37). Other therapeutic options include corticosteroids, azathioprine, or corticosteroids combined with interferon-beta (IFNß) [38]. Furthermore, in a recent study [39] the authors investigated the pathogenesis of myocarditis in a pediatric population, showing that B19V is able to replicate in endothelial cells of small arterioles and venules causing impair microcirculation with the consequence of cardiac myocyte necrosis. In addition, the authors, analyzing the inflammatory responses induced by the virus (as production of IL-1 or IL-6), postulated on the possible role of Interferon-ß or Anakinra for acute B19V-induced myocarditis.

To date, there are no studies that compare these treatment options and immunomodulatory or immunosuppressive therapy remains centre and practitioner specific. In particular, immunosuppressive therapy (prednisone, azathioprine, cyclosporine) is controversial in the treatment of viral myocarditis. Some authors not showed benefit with immunosuppressive agents [40], while other authors showed a significant improvement in left ventricular systolic function with the use of steroids [41] or their usefulness in giant cell and granulomatous myocarditis [42].

Regard the therapy with IFNß, promising results are described in studies conducted of adult population [43] and recently studies on children showed a significant cardiac function improvement in patients treated with an association of IFNß and steroids [38].

However, B19V can cause a devastating myocarditis in children and when maximal supportive therapy does not lead to improvement cardiac transplantation may be considered. Evidences in pediatric population is poor and are present only single-case reports and small series in the literature. For example, Molina et al. [44], in their series of 19 cases of B19V myocarditis reported a high rate of transplantation (42%); Vigneswaran et al. [45] reported in their cohort of 17 patients a death rate of 29% and only one patient underwent heart transplantation. Negative prognostic factors were fulminant myocarditis, ST segment changes or a short prodrome. Further well-designed studies are needed to clarify the role of parvovirus B19 in cardiac disease and the Outcome of B19V myocarditis [46].

### Neurological manifestation

B19V is also associated with neurological manifestations, affecting both the central nervous system (CNS) and peripheral nervous system (PNS). The pathogenetic mechanisms underlying nervous system involvement in B19V infection include direct viral toxicity, dysregulated immune responses with cytokine release, immunocomplex deposition on endothelial cells, and the release of the toxic NS1 protein [47].

CNS manifestations include encephalitis, encephalopathy, meningitis, and stroke. Children with encephalitis often present with convulsions, and approximately one-quarter of these cases exhibit focal neurologic findings [48]. Stroke has also been reported in patients with sickle cell disease experiencing an aplastic crisis [49].

PNS manifestations include neuralgic amyotrophy (inflammatory brachial plexopathy), paresthesia, carpal tunnel syndrome, Guillain-Barré syndrome, and peripheral facial paralysis [50]. CNS involvement is more frequent in children, whereas PNS manifestations are more common in immunocompetent and older patients.

These observations are supported by two reviews. A review by Barah et al. [47] identified 129 reports of neurological manifestations of B19V infection between 1970 and 2012, with encephalitis being the most common CNS manifestation related to B19V. The association with parvovirus infection was confirmed by detecting B19V DNA in cerebrospinal fluid or by positive IgM serology indicative of acute infection. Similarly, a review by Douvoyiannis et al. [51] analyzed 81 cases of neurological manifestations associated with B19V infection, confirming that CNS manifestations are more frequent, with neurological sequelae occurring in 22% of patients. In their cohort, one-third of the patients had altered immunity. Although the authors observed a higher frequency of brain MRI abnormalities in patients with altered immunity, they concluded that there is no significant difference in the risk of sequelae between immunocompetent patients and those with altered immunity.

Currently, there is a lack of clear guidance on the treatment of B19V-associated encephalopathy. However, the use of IVIG and/or steroids may be considered in severe cases, based on a few case reports in the literature that have shown clinical improvement with this therapy. Nonetheless, randomized prospective clinical trials are needed to confirm the efficacy of this treatment regimen.

### Manifestations in transplant recipients

Transplanted children are considered an at-risk population because of their immunosuppressed status [52]. In these patient clinical manifestations of B19V may be atypical and the infection can have severe course. In solid organ transplantation, the common symptoms described are fever (25%) and anemia (99%), while rash and arthropathy are rare because of lack of antigen-antibody complexes. Anemia can be severe or persistent. In the case of unexplained anemia or reticulocytopenia in transplant recipients the diagnosis of Parvovirus B19 infection should be suspected. Other clinical manifestations are pancytopenia or graft loss or dysfunction.

The diagnosis based on the detection of viral DNA, in fact sierological test can be negative due to inadequate or delayed antibody-mediated immune response. Furthermore, high‐level viremia is more likely to be associated with symptomatic disease. The therapy is IVIG at the dosage of a total of 2 g/kg over a period of 2‐5 days, higher daily doses are associated with major incidence of toxicity [29, 53].

B19V infection has also been reported in pediatric allogeneic hematopoietic cell transplantation with high morbidity and potential life-threatening complications. Most patients were treated with success with red blood cell transfusion and intravenous immunoglobulins [54]. Other strategies therapeutic are the reduction of immunosuppressive medication or the use of other immunosuppressive agents as everolimus [55]. Finally, different studies reported other manifestation of B19V infection in transplant recipients, as collapsing glomerulopathy and thrombotic microangiopathy in renal transplant recipients [56] or idiopathic (autoimmune) thrombocytopenic purpura in liver transplantation [57].

### Other manifestations

B19V is associated with a variety of other manifestations beyond its typical presentations. In the liver, B19V can cause a wide range of diseases, from mild elevations in hepatic transaminase levels to more severe conditions such as viral-like acute hepatitis [58]. In rare cases, B19V infection can progress to acute or fulminant liver failure, which can be life-threatening [59]. While there is no specific antiviral therapy for B19V-related liver diseases, treatments such as immunoglobulin, corticosteroids, and cyclosporine have been considered as potential therapeutic options. However, these treatments are generally supportive and tailored to the severity of the liver involvement.

Additionally, B19V has been linked to other renal complications, including glomerulonephritis, nephrotic syndrome or thrombotic microangiopathy [60, 61]. These renal manifestations further underscore the broad clinical spectrum of B19V and the need for careful monitoring and management in affected patients.

## Parvovirus B19 infection in pregnancy

The rate of transmission of maternal B19V infection to the fetus ranges from 17 to 33%, with the highest risk occurring between 9 and 20 weeks of gestation [62]. In most cases, the infection resolves without any adverse consequences. However, the potential effects of B19V infection during pregnancy include:

- **Spontaneous Abortion**: The risk of spontaneous abortion is highest before 20 weeks of gestation, with a rate of 13% before 20 weeks and 0.5% after 20 weeks.
- **Non-Immune Hydrops Fetalis**: The risk of developing non-immune hydrops fetalis varies based on the timing of infection. It is less than 5% in the first trimester, about 10% between 13 and 20 weeks, and less than 1% after 20 weeks of gestation. Ultrasound findings indicative of hydrops include ascites, skin edema, pleural and pericardial effusions, and placental edema.

Routine screening for B19V during pregnancy is not recommended. However, if there is a high suspicion of acute infection, serologic testing is indicated. Recent maternal intrauterine B19V infection is defined by seroconversion from IgG and IgM negative to positive during pregnancy, or by an increase in IgG titers after 2 weeks in previously IgG and IgM positive cases, or by a transition from IgG negative and IgM positive to IgG positive after 2 weeks .

Congenital infection is confirmed by detecting B19V DNA via PCR in fetal blood obtained by amniocentesis (which carries a lower risk of complications for the fetus) or cordocentesis [62, 63].

For pregnant women recently infected with B19V, close ultrasound (US) follow-up is recommended to monitor for the onset of fetal anemia. Fetal anemia is diagnosed by measuring the peak systolic velocity of the middle cerebral artery (PSV-MCA) using Doppler ultrasound [64]. If US findings suggest severe fetal anemia, fetal hematocrit should be determined by cordocentesis.

In cases of unconfirmed anemia, careful monitoring with Doppler ultrasound every 1–2 weeks is recommended for up to 12 weeks after the onset of infection. If severe anemia is confirmed, intrauterine red blood cell transfusion should be performed on fetuses between 18 and 35 weeks of gestation [65]. There is limited evidence on the use of IVIG in pregnancy. One case report described the successful treatment of B19V-infected hydrops fetalis with gammaglobulin injection into the peritoneal cavity, leading to the resolution of fetal anemia and hydrops [66]. However, due to the lack of substantial evidence, this treatment is not recommended at this time.

## Conclusion

Parvovirus B19 is a versatile pathogen responsible for a wide array of clinical manifestations, ranging from mild symptoms to severe, life-threatening complications. The most well-known presentation, Fifth disease or Erythema infectiosum, is typically characterized by a mild rash and other minor symptoms, primarily affecting children. However, B19V clinical impact extends far beyond this, with the potential to cause significant complications such as arthralgia, hematologic disorders like transient aplastic crisis, and adverse pregnancy outcomes, including miscarriages and non-immune hydrops fetalis. B19V ability to involve multiple organ systems - cardiac, neurological, hepatic, and renal -underscores the importance of a comprehensive approach to diagnosis and management. The severity of these manifestations can vary widely, necessitating a range of treatment strategies that may include symptomatic therapies, immunoglobulins, corticosteroids, or other forms of supportive care. Early recognition and prompt intervention are crucial to improving clinical outcomes for patients affected by B19V-related complications. Currently, there is no specific antiviral therapy or vaccine available for B19V, which presents a significant challenge in the management of this infection. However, ongoing research efforts are focused on discovering compounds that can inhibit B19V replication, offering hope for more targeted treatments in the future. Until such therapies are developed, clinicians must remain vigilant in recognizing and managing the diverse and potentially severe complications associated with B19V.

## Data Availability

Not applicable.

## Abbreviations

B19V: Parvovirus B19
CFU-E: Colony-forming units
BFU-E: Burst-forming units
